# Mortality attributable to third-generation cephalosporin resistance in Gram-negative bloodstream infections in African hospitals: a multi-site retrospective study

**DOI:** 10.1093/jacamr/dlaa130

**Published:** 2021-01-19

**Authors:** Angela Dramowski, Gerald Ong’ayo, Andrea M Rehman, Andrew Whitelaw, Appiah-Korang Labi, Noah Obeng-Nkrumah, Awa Ndir, Marcelyn T Magwenzi, Kenneth Onyedibe, Martin Wolkewitz, Marlieke E A de Kraker, J Anthony G Scott, Alexander M Aiken, MBIRA study collaborators, Hilda Mujuru, Emily Odipo, Salim Mwarumba, Neema Mturi, N Claire Gordon, Amadou Diop, Aissatou Thilor Ndiaye, David Shwe, Udochukwu M Diala, Mark Okolo, Hajo Grundmann

**Affiliations:** 1 Department of Paediatrics and Child Health, Faculty of Medicine and Health Sciences, Stellenbosch University, Cape Town, South Africa; 2 Kenya Medical Research Institute-Wellcome Trust Research Programme, Kilifi, Kenya; 3 Department of Infectious Disease Epidemiology, London School of Hygiene and Tropical Medicine, London, UK; 4 Department of Medical Microbiology, Faculty of Medicine and Health Sciences, Stellenbosch University, National Health Laboratory Service, Tygerberg Hospital, Cape Town, South Africa; 5 Department of Medical Microbiology, University of Ghana and Korle-Bu Teaching Hospital, Accra, Ghana; 6 Department of Medical Laboratory Sciences, School of Biomedical and Allied Health Sciences, College of Health Sciences, University of Ghana, Accra, Ghana; 7 Institut Pasteur, Dakar, Senegal and Infection Control Africa Network, Cape Town, South Africa; 8 Department of Medical Microbiology, College of Health Sciences, University of Zimbabwe, Harare, Zimbabwe; 9 Department of Medical Microbiology, Jos University Teaching Hospital, Jos, Nigeria; 10 Institute of Medical Biometry and Statistics, Faculty of Medicine and Medical Center, University of Freiburg, Freiburg, Germany; 11 Geneva University Hospitals and Faculty of Medicine, Geneva, Switzerland

## Abstract

**Background:**

Bloodstream infections (BSI) caused by Enterobacteriaceae show increasing frequency of resistance to third-generation cephalosporin (3GC) antibiotics on the African continent but the mortality impact has not been quantified.

**Methods:**

We used historic data from six African hospitals to assess the impact of 3GC resistance on clinical outcomes in *Escherichia coli* and *Klebsiella pneumoniae* BSI. We matched each bacteraemic patient to two uninfected patients. We compared outcomes between 3GC-susceptible and 3GC-resistant BSI and their respective uninfected controls using Cox regression models.

**Results:**

For 1431 *E. coli* BSI patients, we matched 1152 (81%) 3GC-susceptible and 279 (19%) 3GC-resistant cases to 2263 and 546 uninfected inpatient controls. For 1368 *K. pneumoniae* BSI patients, we matched 502 (37%) 3GC-susceptible and 866 (63%) 3GC-resistant cases to 982 and 1656 uninfected inpatient controls. We found that 3GC-resistant *E. coli* had similar hazard ratios (HRs) for in-hospital mortality over their matched controls as compared to susceptible infections over their controls (ratio of HRs 1.03, 95% CI 0.73–1.46). Similarly, 3GC-resistance in *K. pneumoniae* BSI was not associated with mortality (ratio of HR 1.10, 95% CI 0.80–1.52). Estimates of mortality impact varied by site without a consistent pattern.

**Conclusions:**

In a retrospective analysis, including the use of matched uninfected patients, there did not appear to be an impact of 3GC-resistance on mortality in *E. coli* or *K. pneumoniae* BSI in African hospitals, as compared with susceptible BSI with equivalent species. Better information on the actual use of antibiotics in treating infections in African hospitals would improve these impact estimates.

## Introduction

Antibiotic resistance is a global challenge with major implications for African countries where severe bacterial infections are common, but access to antibiotics is often limited.^1^^,^^2^ Bloodstream infections (BSI) caused by the pathogens *Escherichia coli* and *Klebsiella pneumoniae* are important contributors to neonatal, child and adult morbidity and mortality in Africa.^3–5^  *E. coli* is a common cause of various community-acquired infections, whereas *K. pneumoniae* typically causes hospital-acquired infections.^3^^,^^6^ High proportions of third-generation cephalosporin (3GC) resistance amongst *E. coli* and *K. pneumoniae* BSI have been reported from many African countries, often substantially exceeding the proportions found in high-income settings.^6–9^ Clinically, 3GC-resistance is important as it usually protects bacteria against almost all antibiotics in the widely used penicillin and cephalosporin classes. Furthermore, in most African hospitals, 3GC antibiotics represent the most broad-spectrum class of antibiotics available, so patients with 3GC-resistant infections often receive inadequate treatment. Despite the increasing occurrence of 3GC-resistant BSI in Africa, regional estimates of the clinical impact of this, or any, form of antibiotic resistance on mortality and length of hospital stay (LOS) are scarce.^9^

Methodologically, measuring the impacts of antibiotic-resistant infections is challenging: there is a potential for confounding due to underlying differences in patients acquiring drug-resistant infections as patients with drug-resistant infections are typically older and sicker. To minimize such confounding, a two-step approach can be applied, whereby the impact of having an infection is first determined by comparing infected and uninfected-but-similar patients, and then the additional impact of resistance is derived from comparing these effect estimates between matched cohorts. Studying all these differences gives the most complete description of the impacts of a resistant infection.^10^ The comparison group for measurements of impact of resistant infections can be either susceptible infections of the same type (a ‘replacement’ scenario, meaning that the drug-resistant infection is hypothetically replaced with a drug-susceptible infection) or no infection (a ‘non-replacement’ scenario, meaning that the drug-resistant infection hypothetically does not occur at all). Although most analyses use the former, there is some evidence that some resistant BSI occur under a non-replacement scenario, especially nosocomial infections,^11^ so both should be considered.

Given differences in populations, access to critical care and antibiotic availability, it is unclear whether existing estimates of the impact of 3GC-resistance from high-income settings are relevant for African countries. A systematic review found that very few studies conducted in Africa have examined impacts relating to 3GC antibiotic resistance.^9^ A study in Senegal found that inpatient mortality associated with ESBL-producing Enterobacterales infections was double that associated with ESBL-negative infections, with 4 days additional LOS in survivors.^12^ Single-site cohort studies of BSI in Tanzanian children^13^ and Ethiopian adults^14^ have also found substantial mortality impacts of ESBL-type resistance, though these effects were described in very small groups of pooled Enterobacterales infections.

In this study, we conducted a retrospective analysis of inpatient data from six African hospitals to estimate the impact of 3GC-resistance on mortality and LOS in patients with *E. coli* and *K. pneumoniae* BSI. We attempted to minimize confounding due to underlying disease and bias due to time-dependency and competing events by basing our study design on the BURDEN study, which was conducted in 11 European hospitals in 2008–9.^15^^,^^16^ We hypothesized that 3GC-resistant *E. coli* and *K. pneumoniae* BSIs result in higher mortality and extended LOS compared with 3GC-susceptible infections caused by the same species.

## Methods

### Study setting

Six public hospitals or hospital complexes from six sub-Saharan African countries were invited to participate in the study: (1) Jos University Teaching Hospital, Jos, Nigeria; (2) Korle-Bu Teaching Hospital, Accra, Ghana; (3) Centre Hospitalier National Universitaire de Fann and Centre Hospitalier National d’Enfants Albert Royer, Dakar, Senegal; (4) Parirenyatwa Hospital, Harare, Zimbabwe; (5) Kilifi County Hospital, Kilifi, Kenya; and (6) Tygerberg Hospital, Cape Town, South Africa). These hospitals were purposively selected based on appropriate quality of microbiological data collection and geographical representation across sub-Saharan Africa. Each hospital had on-site laboratory facilities and five had previously published microbiological data on the local profile of antibiotic resistance in Gram-negative BSI isolates.^3^^,^^6^^,^^12^^,^^17–19^ All laboratories participated in national external quality assurance programmes, although the culture, identification and antibiotic susceptibility testing methods varied between sites. Cefotaxime susceptibility test results were interpreted based on standard practice in participating laboratories, using either the CLSI (five laboratories) or EUCAST (Senegal) breakpoints for Enterobacteriaceae*,* as advised at the time of testing. Three laboratories (hospitals 1, 5 and 6) performed routine detection of ESBLs by double disc synergy testing and via the automated Vitek II system (BioMérieux, Marcy l’Etoile, France) (see Table 1).

**Table 1.** Participating institution demographics and identification of the study population

MBIRA study sites	Hospital 1 (Nigeria)	Hospital 2 (Ghana)	Hospital complex 3 (Senegal)	Hospital 4 (Zimbabwe)	Hospital 5 (Kenya)	Hospital 6 (South Africa)
Hospital type	academic, regional	academic, central	academic, central	academic, central	non-academic, regional	academic, central
Number of beds	620	1553	340 (Fann); 120 (Albert Royer)	962	189	1334
National HIV prevalence (adults)	2.9%	1.6%	0.4%	13.5%	5.4%	18.9%
Identification of BSI cases	laboratory records	laboratory records	laboratory records	laboratory records	laboratory records	laboratory records
	(paper-based)	(paper-based)	(paper-based)	(paper-based)	(electronic)	(electronic)
BSI extraction range	2011 – 2016	2016	2012–16	2012–17	2002–17	2010–16
Blood culturing method	Manual	Bactec	Bact-alert (Fann); Manual (Albert)	Bactec and manual	Bactec	Bactec/BacT-Alert
Antibiotic susceptibility testing method	Kirby Bauer	Kirby Bauer	Kirby Bauer	Kirby Bauer	Kirby Bauer	Vitek 2
Approximate blood cultures per month	120	300	75 (paediatric)+65 (adult)	200	350	700
Typical first line antibiotics for community-acquired BSI	ampicillin + gentamicin or ceftriaxone	cloxacillin + amikacin (N); variable (P)	ampicillin + gentamicin or amoxicillin/clavulanic acid	ceftriaxone	ampicillin + gentamicin (P) benzylpenicillin + gentamicin (A)	ampicillin + gentamicin (P) ceftriaxone (A)
Typical first line antibiotic/s for hospital-acquired BSI	ceftazidime or ceftriaxone or ciprofloxacin + gentamicin	cloxacillin + amikacin (N) variable (P)	ceftriaxone or cefixime	ceftriaxone	ceftriaxone ± amikacin (P) ceftriaxone (A)	Piperacillin/tazobactam + amikacin (wards); meropenem (ICU)
Identification of non-bacteraemic controls	ward registers	ward registers	ward registers	electronic admission register	electronic admission register	electronic admission register

BSI, bloodstream infection; N, neonatal; P, paediatric; A, adult.

### Antibiotic treatment practices

Most hospitals used either a 3GC or a combination of ampicillin and gentamicin for suspected community-acquired BSI. Empirical therapy for hospital-acquired BSI varied widely between hospitals (Table 1). No patient-level data on actual antibiotic use was collected in this study.

### Study design

All consecutive *E. coli* and *K. pneumoniae* BSI episodes and their 3GC-resistance phenotype (susceptibility or resistance to cefotaxime) were identified retrospectively from laboratory records using manual record review (hospitals 1–4) or electronic laboratory database extraction (hospitals 5 and 6). Inpatients with laboratory-confirmed monomicrobial BSI with cefotaxime susceptibility results available were included, regardless of patient age or the timing of specimen submission. We defined BSI episodes with blood culturing performed within 48 h of admission as community-acquired, thereafter infections were considered hospital-acquired. Repeat-positive blood cultures within 10 days of the original episode were excluded. Each site investigator selected the longest locally possible time-period within which to identify BSI episodes, aiming to retrospectively collect data on at least 50 consecutive *E. coli* and 50 *K. pneumoniae* BSI.

### Selection of uninfected controls

For each BSI patient identified, two uninfected patients were selected who were matched on age category [neonates (0–28 days), infants (29–364 days), children (1–14 years) and adults (>14 years)], hospital ward and month of admission. An additional matching criterion was that the uninfected patients’ admissions were required to last at least as long as the time-to-bacteraemia for the BSI case (interval from admission to blood culture collection) to avoid immortal time bias.^20^ The uninfected controls were manually identified from inpatient ward registers (hospital 1–3) or using electronic admission registers (hospitals 4–6). If more than two non-infected patients were eligible for matching, investigators selected those with admission dates closest to that of the BSI patient. We included patients with BSI when only a single suitable uninfected control could be identified. For each patient, date of birth, date and ward of admission and date and type of hospital outcome (discharge or in-hospital death) were recorded. For patients with BSI, we additionally collected blood culture date, BSI pathogen and 3GC-susceptibility result.

### Statistical analysis

Following data cleaning, analyses were done in Stata 16.0 (StataCorp, 2019). Outcomes were time to inpatient mortality and LOS, with analyses performed separately for each pathogen. Analysis of hospital inpatient cohorts has the potential to introduce bias and confounding by LOS, co-morbid illness or severity of disease.^15^^,^^16^^,^^21^ The use of multivariate Cox regression models considering competing events, and using time since infection as analysis time for exposed and unexposed patients, is recommended to minimize time-dependent bias.^22^ Cause-specific Cox regression models with robust standard errors were used to evaluate the effect of 3GC-resistance on in-hospital mortality and discharge, measured as hazard ratios (HR). We did not collect data on co-morbid illness. Time at risk commenced on the date of blood culture for the BSI case. For controls, time at risk commenced on the day of hospital stay corresponding to the number of days between admission and blood culture for their matched bacteraemic case. The proportional hazards assumption was assessed using Schoenfeld residuals. The effect attributable to 3GC-resistance was ascertained as the ratio of the hazard ratios for resistant and susceptible parallel cohorts.^23^ We also performed sub-group analysis by site in order to gain an understanding of heterogeneity of effects between sites. We also used Fine and Gray’s extended Cox regression model to simultaneously consider discharge from hospital and death as two possible competing events, and generated the sub-distribution HR for death.^24^

Cumulative incidence of in-patient mortality and discharge from hospital after BSI onset or enrolment of matches was generated accounting for competing risks using the ‘stcompet’ command in Stata.^25^ Calculation of the impact of resistance phenotype on LOS in days was obtained using a generalized linear model with gamma distribution and log link function for positively skewed data. The effect on LOS attributable to 3GC-resistance was ascertained as the predicted marginal mean difference between resistant and susceptible parallel cohorts, with a 95% CI obtained using the linearization method to allow for violations of distributional assumptions.

### Study approvals

Ethics approval and waiver of individual informed consent was granted by the Health Research Ethics Committee at each site. Permissions to access the laboratory and ward registers were obtained from the hospitals’ laboratory and facility managers, respectively.

## Results

A total of 1431 *E. coli* BSI and 1368 *K. pneumoniae* BSI episodes were identified from the six sites (Table 2), although two hospitals (Kenya and South Africa) contributed much larger datasets (88% of the total *E. coli* and 82% of the total *K. pneumoniae* BSI patients). At all study sites, patients paid for blood culture sample processing, except for the South African site where patients did not pay for laboratory testing and in the Kenyan site where blood culturing was funded by research grants. In most sites, these data were collected between 2010 and 2017, except in one site (Kenya), where data collection commenced in 2002. The microbiological prevalence of 3GC-resistance ranged from 13% (in South Africa) to 75% (in Ghana) for *E. coli* BSI (overall 20%) and from 53% (in Nigeria) to 77% (in Senegal) for *K. pneumoniae* BSI (overall 63%; see Table 2 for both organisms). The majority of *E. coli* infections were community-acquired (72%) whereas *K. pneumoniae* infections were mainly hospital acquired (67%) (Table 2). Neonatal and paediatric BSI predominated at hospitals in Nigeria and Ghana, whereas adults contributed most BSI events from the hospitals in Zimbabwe and South Africa, although blood culturing practices were not conducted uniformly across age groups in several sites, so we not interpret these results to represent underlying incidence patterns of bacteraemia.

**Table 2.** Profile of *E. coli* and *K. pneumoniae* BSI episodes

Study sites	Hospital 1 (Nigeria)	Hospital 2 (Ghana)	Hospital complex 3 (Senegal)	Hospital 4 (Zimbabwe)	Hospital 5 (Kenya)	Hospital 6 (South Africa)	Pooled data (all sites)
*E. coli* BSI							
* E. coli* BSI (*n*)	32	56	35	51	507	750	1431
Age distribution EC, *n* (%)							
Neonates (0–28 days)	18 (56.3%)	36 (64.3%)	1 (2.9%)	13 (25.5%)	100 (19.7%)	96 (12.8%)	264 (18.4%)
Infants (29–365 days)	5 (15.6%)	9 (16.1%)	3 (8.6%)	4 (7.8%)	105 (20.7%)	49 (6.5%)	175 (12.2%)
Children (1–14 years)	7 (21.9%)	11 (19.6%)	17 (48.6%)	7 (13.7%)	135 (26.6%)	44 (5.9%)	221 (15.4%)
Adults (>14 years)	2 (6.3%)	0 (0%)	14 (40.0%)	27 (53.0%)	167 (33.1%)	561 (74.8%)	771 (53.9%)
3GC-resistance, *n*/*N* (%)	13/32 (40.6%)	42/56 (75.0%)	22/35 (62.9%)	20/51 (39.2%)	84/507 (16.6%)	98/750 (13.1%)	279/1431 (19.5%)
Hospital-onset BSI^a^, *n*/*N* (%)	6/32 (18.8%)	42/56 (75.0%)	33/35 (94.3%)	29/51 (56.9%)	58/507 (11.4%)	232/754 (30.9%)	400/1431 (28.0%)
Crude mortality rate	5/32 (15.6%)	12/56 (21.4%)	10/35 (28.6%)	8/51 (15.7%)	223/507 (44.0%)	219/750 (29.2%)	477/1431 (33.3%)
Length of stay after enrolment, days (median, IQR)	11 (7–17.5)	5.5 (3–10.5)	7 (6–9)	4 (2–9)	5 (2–12)	10 (4–20)	8 (3–16)
*K. pneumoniae* BSI							
* K. pneumoniae* BSI (*n*)	64	43	64	79	282	836	1368
Age distribution, *n* (%)							
Neonates (0–28 days)	43 (67.2%)	36 (83.7%)	14 (21.9%)	30 (38.0%)	168 (59.2%)	235 (28.1%)	526 (38.5%)
Infants (29–365 days)	4 (6.3%)	2 (4.7%)	13 (20.3%)	23 (29.1%)	44 (15.5%)	92 (11.0%)	178 (13.0%)
Children (1–14 years)	14 (21.9%)	5 (11.6%)	17 (26.5%)	3 (3.8%)	31 (11.3%)	55 (6.6%)	125 (9.1%)
Adults (>14 years)	3 (4.7%)	0 (0%)	20 (31.2%)	23 (29.1%)	39 (13.8%)	454 (54.3%)	539 (39.4%)
3GC-resistance, *n*/*N* (%)	34/64 (53.1%)	31/43 (72.1%)	49/64 (76.6%)	58/79 (73.4%)	182/282 (64.5%)	512/836 (61.2%)	866/1368 (63.3%)
Hospital-onset BSI^a^, *n*/*N* (%)	14/64 (21.9%)	30/43 (69.8%)	56/64 (87.5%)	63/79 (79.7%)	161/282 (57.1%)	590/836 (70.5%)	914/1368 (66.8%)
Crude mortality rate	11/64 (17.2%)	11/43 (25.6%)	25/64 (39.1%)	14/79 (17.7%)	152/282 (53.9%)	276/836 (33.0%)	489/1368 (35.8%)
Length of stay after enrolment, days (median, IQR)	9.5 (5–19.5)	6 (2–11)	6.5 (4–10.5)	7 (3–18)	6 (1–18)	13 (6–26)	10 (4–22)

a Hospital-onset BSI defined as where blood culture sampling was performed ≥2 days after hospital admission.

### Hospital mortality

Crude in-patient mortality for all *E. coli* BSI was high at 33% (477/1442) and varied substantially by country ranging from 16% (Nigeria) to 44% (Kenya). For all *K. pneumoniae*, BSI mortality was similarly high at 36% (489/1368), varying from 17% (Nigeria) to 54% (Kenya) (Table 2). Among fatal cases, the median interval from blood culture sampling to death was 3 (IQR 1–8.5) days for susceptible and 3 (IQR 1–9) days for resistant *E. coli* BSI and 5 (IQR 1–13) days for susceptible and 3 (IQR 1–9.5) days for resistant *K. pneumoniae* BSI.

**Figure 1.**
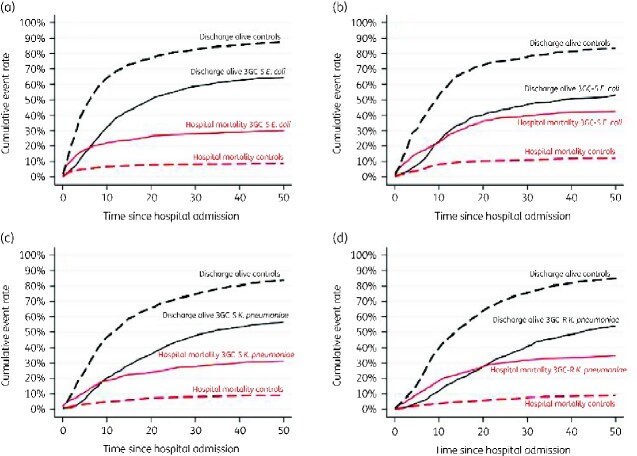
Cumulative incidence of inpatient death and hospital discharge for patients with susceptible (a) and resistant (b) *E. coli* BSI and with susceptible (c) and resistant (d) *K. pneumoniae* BSI, and respective controls. Time since admission is in days.

Figure 1 illustrates the dynamics of the competing outcomes (‘death’ versus ‘discharge’) for *E. coli* BSI [3GC-susceptible and -resistant (Figure 1a and b)] and for *K. pneumoniae* BSI (3GC-susceptible and resistant [Figure 1c and d)] and their respective matched patients. Most deaths in patients with bacteraemia occurred early. Patients with both susceptible and resistant *E. coli* and *K. pneumoniae* infections were discharged at a slower rate than their respective controls, with a stronger effect in the latter.

In the analysis of *E. coli* bacteraemias, 30.9% (356/1152) of patients with 3GC-susceptible infections died in hospital versus 10.0% (207/2056) of their matched controls. Amongst patients with 3GC-resistant *E. coli* bacteraemia, 43.4% (121/279) of patients died in hospital versus 12.8% (70/546) of their matched controls. In the Cox regression model, the HRs for death as compared with the respective controls were 2.82 (95% CI 2.10–3.79) for resistant infections and 2.73 (95% CI 2.29–3.24) for susceptible infections. The overall ratio of HRs for inpatient mortality in 3GC-resistant versus 3GC-susceptible *E. coli* infection was 1.03 (95% CI 0.73–1.46; *P = *0.85, see Table 3).

**Table 3.** Impact of third-generation cephalosporin resistance on in-hospital mortality, discharge and length of stay in *E. coli* and *K. pneumoniae* BSI

	HR (95% CI)			Excess LOS, days (95% CI)
Comparison	Cox model (death)	Cox model (discharge alive)	Fine + Gray model (death)	
R-*E. coli* versus matched controls	2.82 (2.10–3.79)	0.51 (0.44–0.59)	4.10 (3.06–5.48)	1.9 (−1.4 to 5.1)
S-*E. coli* versus matched controls	2.73 (2.29–3.24)	0.54 (0.50–0.58)	3.81 (3.21–4.51)	4.5 (3.1–5.8)
R-*E. coli* versus S-*E. coli*^a^	1.03 (0.73–1.46)	0.94 (0.79–1.11)	1.08 (0.77–1.51)	0.80 (0.59–1.09)
R-*K. pneumoniae* versus matched controls	2.89 (2.38–3.50)	0.47 (0.43–0.51)	4.55 (3.77–5.49)	6.2 (4.5–7.8)
S-*K. pneumoniae* versus matched controls	2.61 (2.03–3.37)	0.51 (0.46–0.57)	3.99 (3.11–5.12)	6.0 (3.9–8.2)
R-*K. pneumoniae* versus S-*K. pneumoniae*^a^	1.10 (0.80–1.52)	0.92 (0.80–1.06)	1.14 (0.83–1.55)	1.01 (0.84–1.21)

Note: for Cox models, these are cause-specific hazard ratios, whilst for the Fine+Gray models, these are sub-distribution hazard ratios.

a Ratio of hazard ratios or difference in marginal means.

In the analysis of *K. pneumoniae* bacteraemias, 33.7% (169/502) of patients with 3GC-susceptible infections died in hospital versus 9.8% (96/982) of their matched controls. Amongst patients with 3GC-resistant *K. pneumoniae* bacteraemia 37.0% (320/866) of patients died in hospital versus 9.7% (160/1656) of their matched controls. In the Cox regression model, the HRs for death as compared with the respective controls were 2.89 (95% CI 2.38–3.50) for resistant infections and 2.61 (95% CI 2.03–3.37) for susceptible infections. The overall ratio of HRs for inpatient mortality in 3GC-resistant versus 3GC-susceptible *K. pneumoniae* infection was 1.10 (95% CI 0.79–1.51; *P = *0.54, see Table 3).

In the Fine and Gray competing risk models for inpatient death, the sub-distribution HRs for all four types of infection were greater than in the corresponding event-specific Cox models for mortality (Table 3). This is because all forms of BSI were also associated with prolonged hospital admission and this model combines the effects of both prolonged admission and increased risk of mortality. However, when comparing 3GC-resistant versus -susceptible forms of both *E. coli* and *K. pneumoniae*, the results were similar to the event-specific Cox models—there was no substantial effect of 3GC resistance in either *E. coli* BSI [ratio of sub-distribution HR 1.08 (95% CI 0.77–1.51)] or *K. pneumoniae* BSI [ratio of sub-distribution HR 1.14 (95% CI 0.83–1.55), see Table 3].

**Figure 2.**
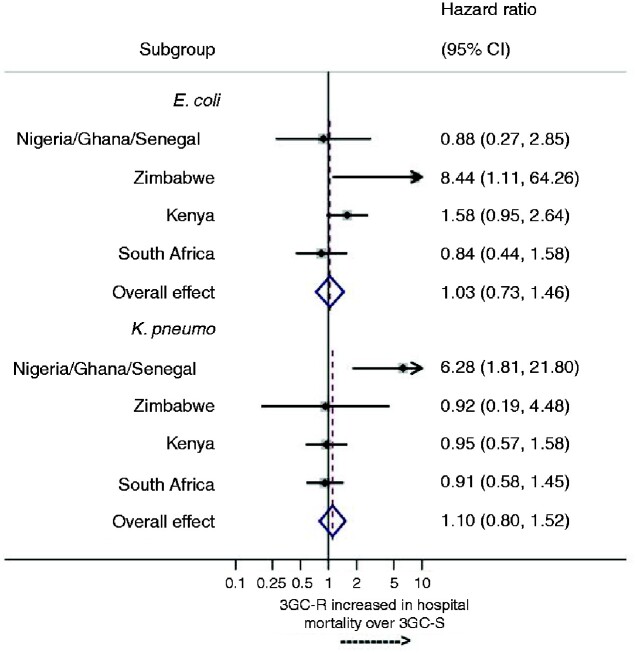
Impact of 3GC resistance on mortality in *E. coli* and *K. pneumoniae* bloodstream infection by country.

Exploratory analysis of site-specific ratios of HRs for the mortality impact of resistant versus susceptible forms of *E. coli* and *K. pneumoniae* are shown in Figure 2. The data for the hospitals in Nigeria, Ghana and Senegal were pooled as these sites had insufficient data to display meaningful site-specific estimates. There was some heterogeneity of site-specific mortality impact for both pathogens, though confidence intervals were wide from the sites providing small datasets.

We considered whether the timing of BSI onset (community-acquired versus hospital-acquired) might have influenced outcomes, as this influenced empirical antibiotic choices in most hospitals, but no substantial mortality difference for either pathogen was noted (data not shown).

### Length of stay

On average, patients with BSI stayed in hospital for longer than their matched controls. Patients with 3GC-resistant *E. coli* infections had an excess LOS of 1.9 days (95% CI −1.4 to 5.1); patients with 3GC-susceptible had an excess LOS of 4.5 days (95% CI 3.1–5.8). Patients with 3GC-resistant *E. coli* BSI stayed in hospital 0.80 times (95% CI 0.58–1.09) longer than those with susceptible infections. Amongst patients with *K. pneumoniae* BSI, 3GC-resistant infections had an excess LOS of 6.2 days (95% CI 4.5–7.8); patients with 3GC-susceptible infections had an excess LOS of 6.0 days (95% CI 3.9–8.2). There was no evidence of longer LOS for patients with 3GC-resistant BSI compared with susceptible BSI for either organism (*P > *0.1).

### Age groups

In sub-analyses with stratification by age (Table 4), there was no clear evidence of a mortality or LOS impact of 3GC resistance in any specific age groups for either *E. coli* or *K. pneumoniae*.

**Table 4.** Impact of 3GC resistance on *E. coli* and *K. pneumoniae* BSI mortality by age category (all sites pooled)

	HR (95% CI)		
Comparison	Cox model (death)	Cox model (discharge alive)	Fine and Gray model (death)
Infants (<365 days)			
R-*E. coli* versus S-*E. coli*	0.50 (0.14–1.86)	1.05 (0.87–1.26)	0.72 (0.40–1.30)
R-*K. pneumoniae* versus S-*K. pneumoniae*	2.31 (0.91–5.91)	0.93 (0.84–1.03)	1.56 (1.01–2.42)
Children (1–14 years)			
R-*E. coli* versus S-*E. coli*	0.75 (0.03–22.41)	1.11 (0.86–1.43)	0.85 (0.37–1.98)
R-*K. pneumoniae* versus S-*K. pneumoniae*	0.10 (0.00–17.21)	0.73 (0.53–1.00)	0.84 (0.28–2.49)
Adults (>14 years)			
R-*E. coli* versus S-*E. coli*	1.77 (0.37–8.36)	0.89 (0.80–0.98)	1.43 (0.87–2.34)
R-*K. pneumoniae* versus S-*K. pneumoniae*	0.96 (0.18–5.10)	1.02 (0.92–1.13)	0.92 (0.55–1.52)

## Discussion

Using retrospective data from six hospitals across sub-Saharan Africa, we produced an estimate of the mortality and LOS associated with 3GC-resistance on this continent. Contrary to our hypothesis, we found no significant additional mortality impact for 3GC-resistance in either *E. coli* (ratio of HR 1.03 95% CI 0.73–1.46) or in *K. pneumoniae* (ratio of HR 1.10 95% CI 0.80–1.52) in a retrospective matched parallel cohort study design. However, in both organisms, when compared against uninfected patients, both 3GC-susceptible and 3GC-resistant BSI did have a large impact on mortality. The same was true for extended LOS; on average, patients with both 3GC-susceptible and -resistant BSI had extended LOS when compared with uninfected patients, but no significant excess LOS could be attributed to 3GC-resistance for either pathogen.

When we performed additional site-specific analyses, for *E. coli*, the mortality impacts of 3GC-resistance at the single largest site (South Africa) and in pooled data from three sites in West Africa were close to 1.0, indicating no effect. By contrast, the mortality impact for 3GC-resistance in *E. coli* at the next largest site (Kenya) was somewhat higher, though still statistically non-significant [ratio of HR 1.55 (95% CI 0.93–2.59)] and higher still at the site in Zimbabwe, albeit with wide confidence intervals (ratio of HR 8.04 (95% CI 1.03–63.0). However, the situation for *K. pneumoniae* was different; there was no suggestion of a mortality impact of 3GC-resistance status either in South Africa (ratio of HR 0.92), Kenya (ratio of HR 0.95) or Zimbabwe (ratio of HR 0.92) but there appeared to be a sizable impact in pooled data from the three smaller sites in West Africa [ratio of HR 6.21 (95% CI 1.77–21.80)]. Thus overall, there was some suggestion of mortality impacts of 3GC-resistance in *E. coli* and *K. pneumoniae* in some of the hospitals, but there was consistently no apparent impact in the single largest patient cohort from one hospital in South Africa. The heterogeneity among sites also suggests the overall pooled estimates might be interpreted with caution.

To explain the absence of mortality impacts in our main analysis, there are several possibilities. First, 3GC-susceptible Gram-negative infections are often resistant to the recommended empirical antibiotic agents, such as ampicillin and gentamicin, which may increase mortality among patients with 3GC-susceptible BSI and attenuate the differential mortality impact for 3GC-resistant versus -susceptible BSI. At the second largest data-contributing site (Kilifi Hospital, Kenya), there is now substantial resistance to the first line WHO-recommended antibiotics among BSI isolates, causing delay in initiating effective therapy for many 3GC-susceptible BSI (personal communication, Dr Claire Gordon). Similar patterns of resistance are seen in Malawi, where surveillance of antibiotic resistance has also been performed over an extended period.^8^ This might explain the similar mortality outcomes between 3GC-resistant and 3GC-susceptible BSI.

Secondly, impacts of 3GC-resistant infections might only be evident when cephalosporin antibiotics are in widespread use and there is limited access to other drug classes (such as quinolones, aminoglycosides or carbapenems). Whilst cephalosporin antibiotics were certainly in use at all the participating hospitals during the study time-periods, retrospectively we cannot quantify their use precisely. Future studies should prospectively gather antibiotic susceptibility data and individual-level antibiotic use, to evaluate the impact of appropriate versus inappropriate antibiotic use for resistant infections in low-income settings.

A third possibility is that the patients included in this study were unrepresentative of all patients experiencing bacteraemia caused by *E. coli* and *K. pneumoniae* in these hospitals. Given the relatively low monthly blood culturing rate in several of the participating sites, there is potential for introduction of selection bias. There is no way to retrospectively correct for this possibility, but future studies should try to enforce standardized blood culture collection criteria and achieve adequate rates of blood culture performance.^26^ This will be challenging whenever patients must self-fund blood cultures costs, as is the case in most African hospitals.

A final possibility is that there is a genuine mortality difference between 3GC-resistant and -susceptible BSI in African hospitals, but that this difference is only small and hence this study was underpowered to detect it against a high background mortality rate. A large study in Thailand showed that pattern of results for the pathogens examined here^27^; it is possible that a similarly small excess mortality exists for these resistant infections in African countries.

We modelled our study design on the BURDEN study, which analysed impacts of *E. coli* and *Staphylococcus aureus* BSI in 11 European hospitals. That study found substantial impact of 3GC-resistance in *E. coli,*^15^ but not for methicillin resistance in *S. aureus*.^16^ There are several important differences between our study and the BURDEN study in the level of data collection. The BURDEN study prospectively gathered data on patient-level variables (such as co-morbidity, in terms of the Charlson Co-morbidity Index) which were used to adjust the relevant models. In our retrospective study, it was not possible to gather this level of detailed information about individual patients from hospital case records, this is a limitation of our study. The resulting lack of adjustment for patient-level variables may also go some way to explaining our findings of no effect of 3GC-resistance in these patients.

An important effect-modifier of the impact of AMR on clinical outcomes, is early appropriate antibiotic treatment. In this study, we were not able to collect individual patient data, but we did have access to local guidelines for empirical prescribing of antibiotics for treating suspected BSI. These guidelines differed widely between participating hospitals. In particular, at the only site with routine use of carbapenems or piperacillin/tazobactam and amikacin for hospital-acquired sepsis (Tygerberg Hospital, South Africa), there was no evidence of mortality impact attributable to 3GC-resistance. This site also contributed most of the patients for this study. For the five other hospitals, therapeutic options for 3GC-resistant BSI were much more limited and access to appropriate antibiotics was restricted to laboratory confirmation of ESBL-production status, substantially delaying the onset of effective therapy.

This study has a number of important strengths. These include: a modest degree of generalizability from the involvement of six widely distributed African hospitals (albeit hospitals with unusually good provision of microbiology services, as compared with other regional hospitals) and representation of all age categories; use of reliable microbiological data from externally accredited microbiology laboratories; focus on a clearly defined form of infection (BSI) in two important Gram-negative pathogens; and the use of an analysis format designed to minimize bias associated with inpatient datasets and time-dependent exposure. The large overall numbers of patients included makes this by far the largest-ever examination of the impacts of antibiotic resistance in African hospitals. Limitations to this work are the retrospective collection of data (which precluded data collection on antibiotic treatment and potential confounders) and the inclusion of small numbers of BSI episodes at four of the six participating hospitals. Whilst we have made a first attempt to supply attributable mortality estimates using data from multiple African countries, generation of further data on the clinical impacts of antibiotic resistance and incidence of infections across the African continent is imperative.

### Conclusions

In laboratory and clinical data collected retrospectively from African hospitals, we did not find statistically significant evidence of additional mortality or LOS impacts associated with 3GC-resistance in inpatients with bloodstream infections caused by *E. coli* and *K. pneumoniae*. However, there were clear impacts of both 3GC-susceptible and -resistant BSI as compared with uninfected patients. Further prospective studies would help to refine these findings and reveal underlying mechanisms, including the role of effective antibiotic therapy in preventing mortality.
