# Erratum to: Mortality attributable to third-generation cephalosporin resistance in Gram-negative bloodstream infections in African hospitals: a multi-site retrospective study

**DOI:** 10.1093/jacamr/dlab037

**Published:** 2021-03-23

**Authors:** Angela Dramowski, Gerald Ong’ayo, Andrea M Rehman, Andrew Whitelaw, Appiah-Korang Labi, Noah Obeng-Nkrumah, Awa Ndir, Marcelyn T Magwenzi, Kenneth Onyedibe, Martin Wolkewitz, Marlieke E A de Kraker, J Anthony G Scott, Alexander M Aiken

*JAC Antimicrob Resist* 2021; **3**: https://doi.org/10.1093/jacamr/dlaa130

In Table 2, the heading of the final column appeared as ‘Hospital 1 (Nigeria)’ but should have read ‘Pooled data (all sites)’. This has now been corrected in the published version of the article.

The Journal apologizes for this error.

